# Art Therapy, Community Building, Activism, and Outcomes

**DOI:** 10.3389/fpsyg.2018.01548

**Published:** 2018-09-24

**Authors:** Holly Feen-Calligan, Julie Moreno, Emma Buzzard

**Affiliations:** Art Therapy Program, Wayne State University, Detroit, MI, United States

**Keywords:** community, art therapy, service-learning, education, action research

## Abstract

This article is a descriptive study of two groups who came together through service-learning: The first group is graduate art therapy students enrolled in a research class, who partnered with six community agencies to help them prepare assignments for undergraduate service-learning students in a subsequent semester. The art therapy research students also assisted the agencies with program evaluation. The second group is the six directors of the community agencies who were preparing for service-learning students enrolled in an art history class titled Art as A Social Practice. Service-learning is an experiential pedagogy where community service is integrated into an academic course, and where the services performed meet genuine community needs. The hyphen in service-learning represents the ideal that both the students and community agencies experience benefits from the relationship, although in reality, it is often the experiences of the students rather than the agencies that receive greater attention in the scholarly research literature. The present article places focus on the community agencies that, in the process of planning for service-learners, made two unexpected requests: First they requested that the service-learners stay longer than one semester, and secondly, they requested assistance with evaluating the effectiveness of *their* programs. This article is about the efforts to respond to these requests through the assistance of art therapy research students. With growing trends in community-based art therapy practice, greater attention to the community agencies where art therapists work is necessary and valuable to art therapy preparation. The present article describes six distinctive communities, illustrating new frontiers of practice. The research students’ experiences and the experiences of the community partners were assessed using qualitative methods that included pre and post-questionnaires, written reflections of students, interviews of agency directors and agency, student, and researcher focus group transcripts. This study will inform other art therapy programs who may want to use a service-learning approach to teaching research. A discussion of the promising practices of service-learning and research, as well as the challenges leads to recommendations for art therapy education.

## Introduction

Growing trends toward community-based health care are expanding the scope of art therapy practice, requiring art therapists to better understand community needs, and to cultivate their unique skill sets for working in community settings (Ottemiller and Awais, 2016). Cultivating skills for community settings can begin in graduate programs, not only in practicum and internship but also in service-learning and research courses. At a time when leading art therapists recommend strengthening art therapy’s research base by scrutinizing and prioritizing issues of importance to the field (Kaiser and Deaver, 2013; Kaiser, 2017), one could argue that strengthening community is a global priority today. Developing research skills, especially outcomes research is also a priority (Slayton et al., 2010). Educators as well are being required to demonstrate evidence that art therapy students develop skills, knowledge and also affective behaviors (Commission on the Accreditation of Allied Health Education Programs [CAAHEP], 2016) appropriate for art therapy practice. Service-learning has the potential to facilitate these priorities.

This article describes new frontiers in community-based art therapy practice and education utilizing critical service-learning to nurture partnerships between art therapy educational programs and community centers and agencies, which has resulted in increased knowledge about community (partner) needs. The article describes a service-learning program and study called ArtsCorps where art therapy research students collaborated with community agencies to assist them with program evaluation and preparation for undergraduate service-learners. Case examples from six agencies provide models for participatory research that add to the art therapy outcomes knowledge base as well as practitioner knowledge (i.e., practice-research cycle).

Service-learning is an experiential learning pedagogy where community service is integrated into the academic curriculum, and where the services performed meet genuine community needs (Howard, 2001). The hyphen in service-learning represents the ideal that both students and community experience benefits from the relationship. Some service-learning studies have been criticized for focusing more on the benefits to students than to the community partners, however (Miron and Moely, 2006; Worrall, 2007). Many service-learning publications describe a commitment to building mutual relationships and to letting members of the community identify their needs, however, what is often missing, is an approach for creating such relationships (Rosenberger, 2000, p. 37). Furthermore, few of the higher education service-learning courses build service-learning projects that are accountable for the results of the service experience on the service “recipient” (Maybach, 1996). Service-learning is used by health professions (Connors et al., 1996; McMenamin et al., 2014) but it has not been well represented in art therapy literature. In art therapy service-learning has not been used in internship (Ierardi and Goldberg, 2014) classes but not to teach research.

The present article describes one part of a larger service-learning study of ArtsCorps. The article explores the experiences of the agency directors who were preparing for service-learners and art therapy research students helping with the preparation. The next section contains definitions of terms used that also provide the perspective of the authors.

### Service-Learning, Research and Other Terms

Service-learning researchers describe different emphases of service-learning. Cipolle (2010) emphasized students’ leadership roles in thoughtfully designed service experiences, with structured time to research, reflect, discuss and connect their experiences to their learning and their worldview (Cipolle, p. 4). She described the potential of service-learning to foster in students a social justice orientation to service, through an examination of issues of power, privilege, and oppression. Sometimes reference to as critical service-learning when the focus is on social responsibility, critical community issues and as a method for problem solving (Fenwick, 2001; Mitchell, 2008).

Service-learning shares qualities with action research, a term coined by Lewin (1947), to refer to problem solving strategies that encourage academic researchers and community members to work together to identify and analyze community problems, find solutions to those problems through research and test those solutions in the community (Harkay et al., 2000).

The emphasis on action research recognizes that service itself can and should be a knowledge-generating activity. When combined with appropriate analysis and dissemination of findings, it can help guide subsequent practice in a variety of settings as it works toward solving specific community problems (Harkay et al., 2000, p. 113).

From this perspective, service and research are intertwined in the process of exploring and responding to community problems, especially when the best ways to solve a community problem are still emerging.

Whyte (1991) used the term participatory action research to highlight the role of both scientists and lay persons as critical to the process of knowledge acquisition. He advocated for collaboration in all aspects of the research process, from the design to the results and discussion of action implications. CBPAR is community based participatory action research.

Community based research (CBR) is a term used by Stoecker (2008) which expands on the practice of service-learning to include research. CBR draws on the research skills students are learning to address community generated research questions (Stoecker, 2008). CBR relies on the community’s participation in research, and should result in the ability of the community to gather and use knowledge relevant to them. This method and philosophy reduces the university’s power and control over knowledge production by recognizing community partners as contributors of knowledge in the service-learning relationship and as partners joining together with the university to problem solve together to improve quality of life.

Consistent with Freire’s (1970) “pedagogy of the oppressed” (where the educational strategies raise consciousness of economically disadvantaged groups so that they could define and articulate their own problems and collectively seek solutions), critical service-learning involves students’ raising consciousness of their privilege and the power differential.

As Harkay et al. (2000) write, “action research is an appropriate model of service-learning because it combines the best elements of democratic cooperation, methodological rigor and service. It also combines the interests of students, community and faculty oriented toward the goals of community development and democratically created social change” (p. 115).

Several art therapists have written about expanding practice to community settings (Timm-Bottos, 2006; Kapitan, 2009). The term community-based art therapy used in this article is defined broadly as “a theoretical framework that emphasizes community empowerment rather than a focus on individual psychodynamics, and provides a vehicle for community strengths and needs” (Golub cited in Ottemiller and Awais, 2016, p.144).

In this study, the faculty chose service-learning philosophy and pedagogy because of its emphasis on social responsibility, its respect for and value of the knowledge of the community partners, and the opportunity to explore problem solving together, ultimately strengthening relationships. These problem-solving strategies and collaboration qualify the art therapy students’ activities as action research and service-learning. In an attempt to clarify the two classes or courses of service-learners described in this article, the art therapy research service-learners are referred to as ATRSL students. The service-learners from the art history class will be referred to as Art History service-learners (AHSL) students. Last, in this article community partner is used interchangeably with agency director.

### ArtsCorps

The university in which our art therapy program resides, “Midwest State University” is a public, urban university with Number 1 rankings of the Carnegie Foundation in research and community engagement. ArtsCorps was established as one of the university’s community engagement initiatives by faculty members in the arts whose vision was to strengthen local arts programs, especially because of the reductions in art education in the city’s public-school system. Given the university’s commitment to the city, the faculty believed the university’s arts departments should be supporting local arts programs. ArtsCorps sought to support local arts agencies through connecting them with volunteers who could assist with special projects, and with service-learners who could partner with the agencies over a longer term, working together to address mutual goals and concerns.

Art therapy students had participated in service-learning assignments and internships at a variety of agencies over the years, which resulted in positive gains to all parties, but by-and-large, the gains had not been systematically documented. We wanted to document students’ experiences, as well as the experiences of the agencies as they host and mentor students. We wanted to know the value students added to the agencies. When an internal university arts and humanities research grant competition was advertised, the author and other arts faculty who had previously taught courses with service-learning assignments invited six directors of community agencies who had in the past hosted our service-learners, to partner with us on a proposal to study the impact of service-learning on students and community partner agencies. Our proposal was funded, and this allowed us to hire two graduate assistants (GA)s (the second and third authors), and in addition to provide the agencies with modest participation stipends.

Although the ArtsCorps study extended 5 years as we added community partners and service-learning courses, this article covers only the first year/beginning phase of the research study, which was devoted to studying the experiences of community agencies, and service-learners from art therapy research classes. Limiting the article in this way focuses attention on the relationships and sense of community that developed between the agencies and the art therapy program. The opportunity to collect some data from the agencies’ constituents has contributed in a small way to understanding art therapy outcomes. A concluding discussion explores art therapy research and service-learning and the implications for practice and education.

## Research Procedure and Methods

The agency directors, faculty and research assistants began meeting together to plan our research study, which was carried out in accordance with the University’s Behavioral Institutional Review Board, Human Investigation Committee. Students and agency directors gave written informed consent in accordance with the Declaration of Helsinki. Information sheets describing the study were provided to agency constituents. The six agencies were: (1) a “soup kitchen” that had an after school art therapy program for children; (2) a children’s hospital that used community volunteers to staff their children’s art program; (3) a residential substance use treatment program for homeless men; (4) an outdoor street art installation where art and recycling classes were offered to school age youth; (5) an organization that sponsored a fund raising race for cancer research; and (6) an art program for people with physical and cognitive disabilities.

At first, the faculty wanted to study students’ and agencies’ experiences with service-learning in an Art History class titled Art as a Social Practice, where participation in service-learning at one of the six agencies would be the main assignment. This course was scheduled for the winter semester, allowing time in the fall to prepare for the AHSL. The faculty also had planned to enlist students enrolled in an art therapy research class to assist with some basic data collection. However, the plan was modified when in a preliminary meeting, the agency directors expressed their desire for AHSL placements to extend beyond one semester, due to the time and effort on their part to train students. They also voiced a need for assistance with assessing *their* programs and their programs’ impact on their constituency groups. Ordinarily in planning for service-learners, the community partners provide some general ideas of how service-learners could contribute to their agencies, and then the specific contributions are negotiated, depending on the capacities and interests of the students in the class. Although the agencies expressed a desire for research assistance, we were not sure whether the art history students had research expertise, and thus we began to explore other options.

Because service-learning assignments were connected with semester-long courses, the university calendar seemed to be an insurmountable obstacle. Yet, as the faculty considered how the agencies’ needs could be met, we realized the art therapy research students typically did enroll in two consecutive semesters: in a research methods class followed by a master’s project class. We wondered whether the research students could provide some greater continuity to the agencies if they had a two-semester presence, and, since the agencies expressed interest in program evaluation, whether the research students could acquire the research knowledge they needed by assisting the agencies with their research agendas. Ultimately, both agency directors and university faculty decided this was a worthwhile option to try: The research course would be taught from a service-learning perspective, meaning that the role of the ATRSL students would be negotiated with the agency just as any other service-learning relationship, so that the capacities and strengths of all parties could be utilized to meet the needs of all parties. The ATRSL students would study the agencies, searching for opportunities that the AHSL students could undertake in the subsequent semester. The ATRSL students would consider how they could function as liaisons who could assist with preparing the AHSL students, saving some time and energy on the part of the agency directors. The ATRSL students would also assist the agencies with evaluating their programs as the major assignment in their research course. As research students the ATRSLs were learning about service-learning as well as an action research.

The agency directors were asked to begin thinking about their research questions and what they wanted to know about their programs. The syllabi for the research classes were modified so that the students enrolled could investigate what became the third area of inquiry in the ArtsCorps study: assisting agencies with assessing their effectiveness. Program evaluation was one of the research methods taught in the research courses, so this year, the students had an opportunity to participate in actual program evaluations. In sum, our three overarching research questions were:

(1) What are students’ experiences as service-learning participants, and what are the learning outcomes of their service-learning experiences?
(2) What are community arts organizations’ experiences with service-learning students, and how does the involvement of service-learning students affect the overall effectiveness of their programs?
(3) Can/how can service-learners support community partners to assess their programs’ effectiveness?

Because the faculty and the agency directors each had a stake in the success of the service-learning projects, we were all co-researchers using a participatory action research approach. The faculty, graduate assistants and agency directors met in three focus groups during the year, and in addition to these meetings, we were in regular email communication.

The art therapy research syllabi were developed in consultation with the community partners. The first of the two research courses was planned to focus on the students’ learning formative evaluations that they could carry out in the agencies, in teams. The purpose of the formative evaluations was to determine the strengths as well as potential areas of need that could be addressed by AHSL students from the *Art as Social Practice* class scheduled for the subsequent semester, as well as to explore how best to assist the agencies with their program assessments. Required readings in the research courses covered service-learning, community art programs, community based participatory action research and program evaluation. **Table 1** shows the research activities and timeline of these activities.

**Table 1:** Research description and time table.

Course	Pre-fall semester	Fall	Winter
Research 1	• Review syllabi for research classes • Contact enrolled students to describe the ArtsCorps Research foci, and begin to generate interest • Confirm agency participation • Schedule agency focus group for service-learning course planning purposes • Finalize selection of service-learning assessments, and/or create assessments • Complete IRB application • Advertise for student assistants to be hired beginning fall	• Students, faculty, and graduate assistants complete IRB training modules • Consent students and agency directors • Administer pre-SL assessment of research students and agency directors • Students in research class learn basic research methods and begin data collection strategies including formative evaluation of participating community agencies • At end of semester, provide agencies with copies of the formative evaluation. This will include recommendations for service-learning assignments and recommendations for research projects for research students in semester II • Administer post assessment for students not continuing next semester • Schedule end of semester focus group, and check in for next semester	

Art History Course: Art as a Social Practice		• At end of fall semester, students enrolled in Art History (AHSL) course will be introduced to the suggested service-learning assignments identified in Sem. 1	• AHSL students will consent to participate in the ArtsCorps Research • Students complete pre-SL Assessment

Research II		• Schedule end of semester focus group	• Research assistants and students in Research II course collect baseline data of students in Art History course (AHSL students) • ATRSL students continue to work with agencies on their research agendas • Schedule post-assessment of students and agency directors

### Data Collection, Research Tools and Analysis

To assess the experiences of the agency directors and the ATRSL students, we collected qualitative data that included pre and post-course questionnaires, students’ written reflections at the end of the semester, interviews of the agency directors and focus group transcripts. The questionnaires were developed by the university researchers to capture the information necessary to plan the course and evaluate learning, supplemented with some questions from standard service-learning assessments that were relevant to our particular research questions.

Each ATRSL student completed a questionnaire at the beginning of their research course to determine a baseline understanding of their experiences with service-learning, knowledge of research, and attitudes about community service. They also completed questionnaires at the end of their research course aimed at exploring what they learned. Additionally, students were asked to submit a short paper reflecting on their service-learning research experiences.

The agency directors completed pre and post questionnaires as well. In addition, their focus group attendance was recorded by a notetaker or was audio recorded.

The agency directors were also interviewed by the research students as part of their research of assignments. **Table 2** lists the data collected.

**Table 2:** Data table.

Data describing:	Type of data:
Research students	Pre-research class questionnaire
	Post-research class questionnaire
	Reflective paper
Agency director	Baseline or preliminary (pre) questionnaire
	Post questionnaire
	Interview
	Transcripts from focus groups
Agencies	Other data collected by ATRSL students • document research • observation • focus groups

The data collection was overseen by the research director (first author) and the two graduate assistants (second and third authors). Researchers met biweekly to discuss ongoing data collection and emerging findings. Qualitative methods were used to search for themes among the questionnaires, interviews and reflections. The NVIVO Qualitative Analysis software assisted with data management, primarily to corroborate themes we thought were emerging. Each set of data was uploaded into NVIVO so that we could search for frequently used terms (to facilitate theme or node identification). We also searched for terms pertinent to our research questions, e.g., art based service-learning, agency experiences, evaluation, outcomes, etc., to help us identify themes. The main benefit of NVIVO was its ability to code multiple sets of data and to reference the source of the coded data for the researcher’s ease of retrieval.

## Research Activities

### Art Therapy Research Students

Prior to the course commencement, a description of the course was posted to the course’s online announcement page, in order to inform students about the plan for the research courses this year. That way, students who were not interested in participating in ArtsCorps research could wait until the following year to enroll the research courses.

As stated, the ATRSL students completed a preliminary questionnaire to obtain baseline measure of their research knowledge. All students completed the questionnaire and participated in all class activities, but only the questionnaires of those consenting to be study participants became part of the data collected. A faculty member not involved in the study administered the consents, and the instructor of the class (the author) did not know who had consented to participate until after the end of the study. A total of 16 art therapy students registered in the research classes during the two semesters; three of these students were in both classes. Twelve students consented to be in the research and there were nine completed sets of pre- and post-questionnaires.

Following consenting the students, the first classes were devoted to learning about the participating agencies through researching their websites and program materials. Basic research topics were covered, with a focus on program evaluation and CBPAR research. The data collection strategies including interview, observation, questionnaire, and document research, and the analysis of data through and using triangulation in the analysis of data, provided the ATRSL students with opportunities to practice these research methods. The course readings covered projects similar to the ones we were undertaking (e.g., Jones, 1988; Worrall, 2007; Puma et al., 2009).

Baseline assessment or formative evaluation (Fitzpatrick et al., 2004) of the needs, strengths, goals, and missions of the organization was the major assignment. In the first semester. Because the primary purpose of a formative evaluation is program improvement (Fitzpatrick et al., 2004) it was an appropriate method to determine the options for best use of the AHSL students, and also to explore possibilities for assisting the agencies with outcomes evaluations of their programs. To conduct a formative evaluation involved researching the agencies’ publicly available literature from their websites or brochures and other documents; observation of its programs, and interviews with the agency director or other personnel.

Although the goal was to ask ATRSL students to choose an agency and to work together as a team, there weren’t enough students who enrolled in the first semester for everyone to work in teams. To attract more students, we opened a section of the master’s project research class (the research project class for students who had already had a methods class) to run concurrently with the research methods class. We ended up with enough students so that three agencies had two students; three had one. Despite the challenges, the students learned how to triangulate data to search for patterns and themes, and then to write their formative evaluations. An oral report was presented to the agency directors in the class on the last day of the semester, and all directors were given final written formative evaluation reports. These reports contained recommendations for the AHSL students, and research recommendations for outcomes assessments, that could begin the subsequent semester with their research ATRSL students.

In addition to the potential projects for the AHSL students in the second semester, the formative evaluations also revealed potential further direction in terms of research with which the ATRSL students could assist in the second semester: Three agences expressed interest in evaluating specific programs they offered. The Art Street Project expressed interest in evaluation of a program they called E^2^CA: Environment and Education, Community and Art; the children’s hospital expressed interest in evaluating their “healing arts-in waiting room” program; and Recovery House requested assistance with evaluation of whether or how group art projects promote team building. The soup kitchen requested assistance with searching for literature that would inform their peace curriculum. The Special Arts was determining how to proceed with their research needs, and the cancer race, as scheduled during the second semester was focused on that event. In the second semester the ATRSL students assisted with these activities through participant-observation at the sites, interviews of the directors, and through focus groups with staff and/or adult program constituents. **Table 3** lists these projects.

**Table 3:** Projects Completed by Art Therapy Research Service-Learners in Conjunction with ArtsCorps, Semester 2.

**Evaluation of Service-learning Experiences of Art History students** Students conducted a literature review of *service-learning in higher education*, and analyzed (AHSL) student experiences with service-learning using pre questionnaire/baseline assessment data, analysis of student blogs and a post/end-of semester questionnaire. They also helped interview agency directors.
**Summative Evaluations of select agency programs** *Children’s Hospital*: Students worked with Children’s Hospital to create, then refine / revise an instrument to evaluate children’s satisfaction/experiences with the hospital’s healing arts group participation. *Art Street:* Students helped with program evaluation of the pilot E^2^CA program. The evaluation protocol included collecting questionnaires from child participants, observations of the program and teacher interviews. *Recovery House:* Students assisted with evaluating the *Yes, We Can* mosaic project. They contributed to the development of the “pre” and “post” assessment of data investigating the extent to which the art form fostered team binding, and they were participant observers.
**Program Proposal for the Soup Kitchen** An ATRSL worked on art therapy program development for the soup kitchen, at their request. This entailed a literature review on peace curricula and interviews of art therapists in similar programs. A program was proposed in writing that integrated peace curricula.
**Grant for Recovery House** An ATRSL worked with Recovery House personnel to write a grant for funding for an art therapy position.
**Historical research/ literature reviews** An ATSL student researched: *What programs exist that are similar to Arts Corps*? A second student researched *What are programs similar to ArtsCorps?* A third student researched *Assessment in community agencies with arts programming* through a literature review.

### Agencies

As stated, the agency directors completed preliminary questionnaires asking what they wanted in a service-learning student and types of projects they were envisioning or needed help with. During the first semester, the ATRSL students interviewed the directors and begin the process of gathering data at each site. The agency directors attended three focus groups with the faculty and graduate research students. At the end of second semester, the agency directors completed a second evaluation about their experiences with service-learners. Because the article focuses on the ATRSL students, the agencies’ experiences will be limited to their work with these service-learners.

The following describes the six agencies using pseudonyms, and a short description of the findings from the formative evaluation and second semester research activities, which were identified through the formative evaluations. Following the agency descriptions is a summary the findings of the agency directors and the ATRSL students. A list of the projects of the AHSL students is in **Supplementary Data Sheet S1**.

#### Recovery House

Recovery House provides residential substance use treatment for homeless men. The aim of Recovery House is to provide the resources necessary for men to become self-sufficient, while recovering from substance addiction. At the time of this project no formal art therapy was part of their program, however, an art therapist had previously been employed there, and artworks and poetry from her tenure were still displayed on bulletin boards. Although the budget prevented replacing the art therapist when she left, Recovery House used artists-in-residence to provide some arts programming. The men were fond of the mosaic tile murals they created with the assistance of the artists and which were displayed in the front lobby, on an outside wall, on outdoor benches and on garden stones. One mosaic in the front lobby was titled “Bridge over Troubled Waters,” as a metaphor for their treatment. The purpose of the formative evaluation at Recovery House was to become familiar with programming currently offered to residents, to understand how the art programming enhanced the treatment program, and to learn about the needs of Recovery House that could be addressed by a service-learning student.

The formative evaluation was conducted through collecting information from the program’s website, brochures and annual reports, a tour of the facility, interviews with the program director and other administrators, an exhibition of art of current and previous Recovery House residents, and conversation with current residents about the programs.

The ATRSL students were told by the residents that initially most wanted nothing to do with art, saying things like “I never did like art,” or “art… and therapy? They don’t really go together!” But eventually the men began to enjoy the art; some used the art making time to make gifts for their families, which made them feel good. One man described his experience with art as “taking [him] to another world”; others experienced a “child-like feeling,” or just being relaxed. Referring to the Bridge over Troubled Water mural, one man said “a lot of us came together,” noting the camaraderie that developed in the process of art making. Men who previously felt hopeless were now praised and encouraged to continue artistic efforts.

Together, the focus group, staff interviews, and the facility that prominently displayed the art indicated that the art programming at Recovery House had positively influenced the men and assisted in their recovery from addiction. The ATRSL student researchers recommended the goal of hiring another art therapist to provide therapeutic programming, in addition to the existing recreational/leisure art groups. The ATRSL students determined that an AHSL student could also support the art programming by initiating new arts programming or working closely with the current art studio volunteers to allow them to do more. Other ideas included enhancing the exhibits there or looking for venues outside Recovery House that could promote advocacy or serve as public awareness about recovery and reduce the stigma about drug addiction.

**FIGURE 1:**
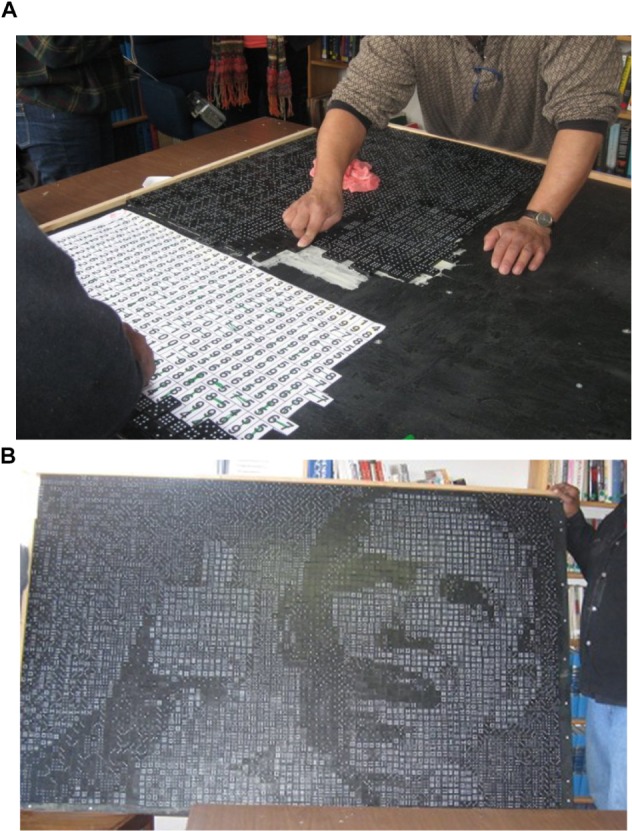
**(A,B)** Recovery house. Creating the domino mural **(A)** and the finished mural of President Obama **(B)**.

Observing the creativity of the population – the interest in mosaics especially, the Recovery House director requested assistance with evaluating whether or how group mosaic projects facilitated “team building,” noting that it is sometimes difficult to become invested in the treatment program until individuals feel a sense of belonging in the community. Additionally, participation in 12-Step recovery groups is an important facet of long term recovery. If comfort with such group experiences could be developed early in treatment, the director hypothesized, this would positively impact recovery in the long run. Furthermore, the director thought that if art making could be implicated in the process of team building, this evidence would be instrumental for future grant applications. The group of Recovery House residents voted to use their stipend from ArtsCorps participation to purchase a grid pattern designed by world-renowned mathematician, Robert Bosch that diagrammed a domino mosaic portrait of former U.S. President Barack Obama. The men co-created the domino mosaic, and their process was evaluated by the ATRSL students, who searched for evidence of team building (**Figures 1A,B**).

In the second semester, the ATRSL students developed a summative evaluation protocol using participant observation of 11 domino mosaic sessions over 26 days; pre and post-mosaic surveys completed by participants; daily written participant self-assessments, a culminating focus group, and interview with the agency director. Throughout the 11 sessions, service-learners observed impulsivity (gluing before reading all instructions), frustration (bumping the board and needing to start over again), and competition vs. cooperation (individuals vying for leadership), but also that eventually the group collaborated and worked together as a team. The men described their process as “challenging … hectic at first… all chiefs and no Indians.” They identified “gravitat[ing] toward what they wanted to do and keep[ing] each other in check.” Most were “loners” at first but the mosaic activity facilitated interaction: “You see another part of people… We knew of each other but with this we came together.” About half the men indicated that they enjoyed working as a group and that it positively impacted their mood. The group was relaxing as well as stimulating, with exposure to new ideas. The participants also gained personal awareness, evidenced by such comments as: “I found out I am stubborn… I can’t draw” or “I can do things… I didn’t have to be so depressed today…. I learned to be self-sufficient…”

**FIGURE 2:**
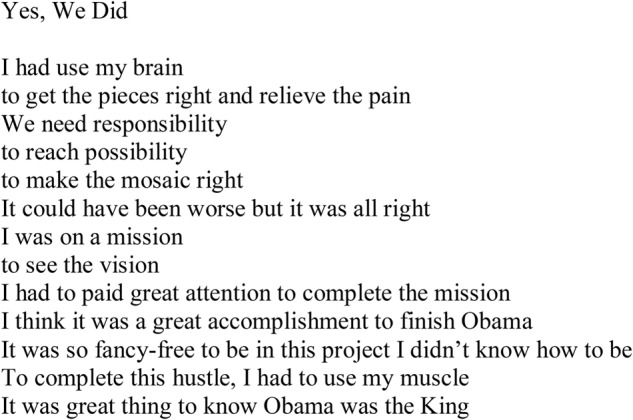
Yes, We Did poem co-written by participants.

Partially because of the interest in poetry, and also to use an art-based method, the focus group was structured so that the men could write a poem to summarize their mosaic experience. The service-learners created a word bank with words heard during the art making sessions. The men were encouraged to use the words to co-create a group poem, they titled, “Yes We Did” (**Figure 2**) an homage to former President Barack Obama’s slogan, “Yes We Can.”

#### Art Street

**FIGURE 3:**
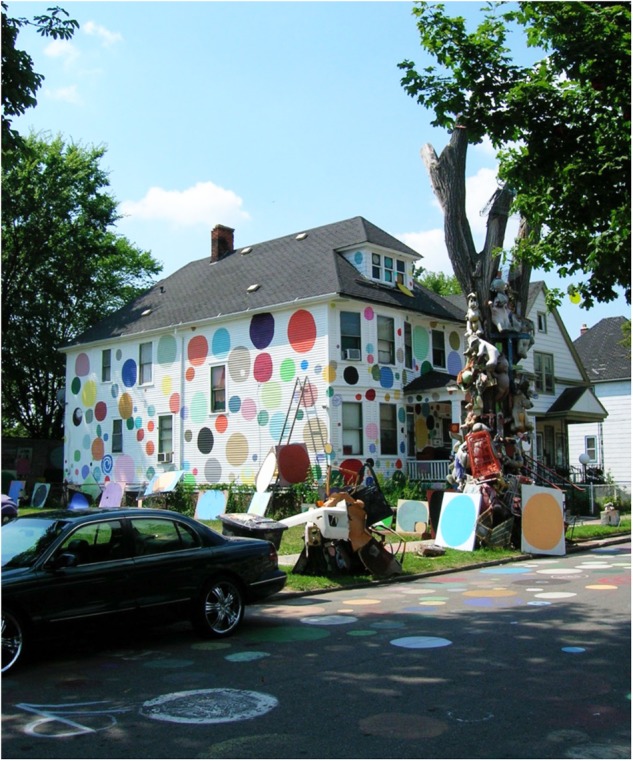
A view of art street.

Initiated by a Detroit artist in 1986, the Art Street Project is literally an outdoor art installation covering a residential street, comprised of discarded objects (stuffed animals, shoes hanging from trees, television sets, car parts, etc.) assembled in ways to make social statements, combined with large paintings or sculptures by the artist (**Figure 3**). Art Street has been described as a public art movement and is the third most visited cultural tourist site in the city, attracting over 200,000 visitors annually. It is categorized as a non-profit art center with a mission to inspire people to appreciate and use artistic expression to enrich and improve their lives, and to improve the social and economic health of the neighboring community. The project continues to expand and build momentum by developing new programming such as a children’s art program called Education, Environment, Community and Art (E^2^CA), where, in the fall and spring, children learn to make art and care for their community environment, and in the winter work inside the studio of the founding artist. E^2^CA was established through a grant to provide a comprehensive program that helps rectify the lack of art education in city public schools and at the same time teaches children the value of the environment and community. In addition to the E^2^CA, Art Street offers public festivals, tours, workshops, and a young artist mentoring program. Although offering art education to children through access to Art Street has always been a part of Art Street’s mission, the E^2^CA program is the most extensive program to date and a big step toward bringing the Art Street philosophy into public awareness. Thus, Art Street was interested in understanding its impact.

The E^2^CA program endeavors to engage and educate students to take responsibility for their home and their community in creative and resourceful ways. City schools can request to have the E^2^CA as part of their curriculum. The program begins with an introductory presentation and workshop at the participating schools facilitated by trained docents from Art Street. This presentation provides an overview for the teachers and students, who are then taken on an interactive guided tour of the Art Street site. The tour includes a personal visit with the artist-founder at his studio. The teachers at participating schools are provided with a seven-week, arts-based lesson plan for grades 3, 7, and 11 to implement within their standard curriculum.

Art Street wanted to measure the impact of E^2^CA on the children. The formative evaluation was designed to provide Art Street with useful information about factors that influence the implementation of the E^2^CA program and how they could collect useful data that would help improve the program. Interviews were conducted with the executive director of Art Street, her assistant, and the program coordinator of the E^2^CA program. In addition, the ATRSL students took a tour of the street and observed the reactions of various visitors also present. A review of program materials was also completed. The results of the formative evaluation recommended collecting multiple sources of data from the E^2^CA constituents: students, parents, teachers, school principal, the E^2^CA program coordinator, and Art Street docents in order to look for evidence of the students’ learning and applying the program concepts.

**FIGURE 4:**
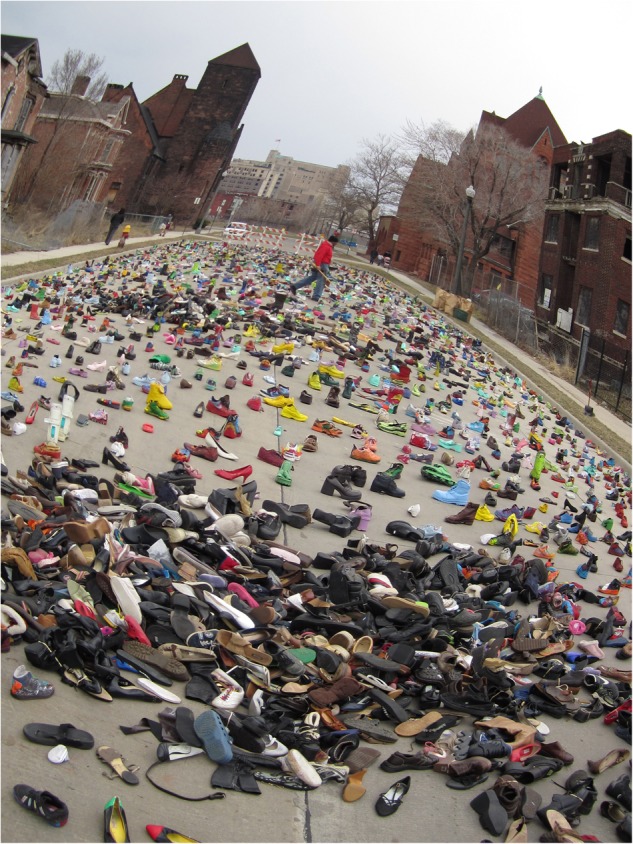
Art Street’s Artist’s exhibit, Streetfolk. Photo: Michelle Figurski.

Potential responsibilities for AHSL in the next semester were recommended to include observation of the E^2^CA classes, and/or to assist with other aspects of the evaluation. For other AHSL students, the Art Street artist was preparing for a new installation on homelessness, and he would need assistance with that (See **Figure 4**).

During the second semester, two new ATRSL students followed through with the plan to evaluate the E^2^CA program, beginning with reading the formative evaluation written in the first semester, and developing questionnaires for students and classroom teachers based on the stated objectives of the program:

Learn new ways to improve one’s community without the use of expensive or precious materials.
Learn the principles of how to reuse and recycle.
Practice community building in school through collaboration and teamwork.

The questionnaires were designed to be administered to the students and the teachers at the beginning, at the mid-point and at the end of the seven-week program.

Over 120 surveys were collected from students at the two participating schools, the two teachers and the Art Street E^2^CA program coordinator. The survey responses from students and teachers, observations in the classroom and on-site and in-studio, indicated that some of the students involved in E^2^CA understood the principles of using art and recycling to improve a community, and learned various facts about the Art Street art and its history. The individual responses from students, particularly when verbalized, provided stronger evidence of their learning. However, because some students did not respond to written or verbal questioning, it is unknown whether they took away knowledge from the program.

As this was the first year of the program, the evaluation continued by ATRSL students in the second semester provided more in the way of formative data (than summative data) for program improvement. For example, an interview with one of the teachers who was asked, “How has the students’ learning been impacted?” responded, “They have more awareness about recycled art, however, the classroom is currently doing nothing to promote recycling efforts.” The teacher has implemented the program in school by “classroom discussion.” This sort of information was useful to know in order to consider ways to improve the program. The elementary students did seem to learn something about art, e.g., “That you can make anything if you put your mind to it… It’s more than just art… Some art makes us remember the past” were a few of the comments, but learning about art was not one of the program objectives. This information was also useful to Art Street as evidence of what children did learn, that perhaps could be integrated into the E^2^CA program to enhance it.

### A Children’s Hospital

**FIGURE 5:**
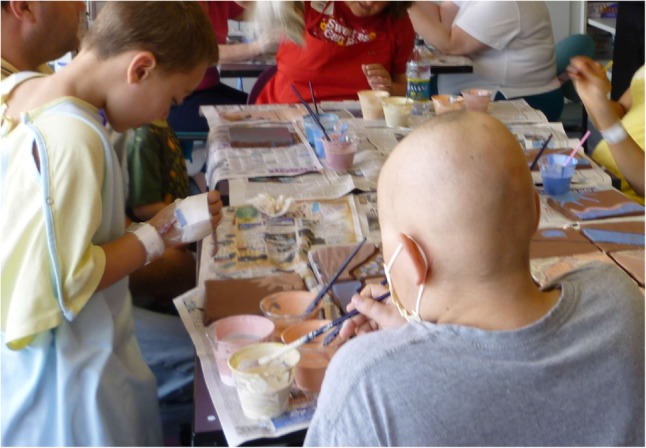
Children’s hospital art group.

The children’s hospital employs one paid staff person in a role titled, Arts Advisor, who coordinates volunteers who facilitate several arts initiatives that include art making in waiting rooms, offering art projects in children’s rooms at their bedsides, and group art projects led by community organizations, such as the city art museum, local pottery studios, or scout troops (**Figure 5**). The Art Advisor sought information to determine whether the volunteers were being used most efficiently or whether she could better maximize her resources to improve services. Of particular interest was the waiting room art making program in which art activities were offered to children and their families while awaiting their medical procedure. The Art Advisor was curious to know whether making art before a medical procedure helped to reduce anxiety among the children and their families.

The formative evaluation was conducted by one student who completed three interviews with the Art Advisor, and three observations of children’s waiting room art sessions (facilitated by artists and other additional volunteers), including one session in which she participated. The ATRSL kept written reflections of her observations and cycled back to the Art Advisor with questions about her observations and her reflections. During the observations the student noticed that the volunteers did not appear to have direction or goals, but rather they were present and interacted with the children. In her reflections the ATRSL noticed the dynamics or interaction between participants and artist/student-volunteers, the therapeutic environment established and the consistency of approach of the volunteers; the goal of each group, how the arts seemed to help each person, whether they appeared to reduce anxiety; and how the arts were viewed by the hospital as a whole.

The ATRSL observed children from age one through adolescence attending the art making sessions along with their parents and siblings. She observed assigned topics in art making, free choice art making, group art projects and socializing during the art making sessions. She observed different roles of the artist and non-artist volunteers. The artists provided a creative art project while the non-artist volunteers offered drawing sheets. There was a lot of movement of the children being called back and forth to see the doctor, so that the art making could last from 30 min to 2 h.

The ATRSL thought that in order to determine how to improve services, a survey could be designed with input from constituents: the volunteers, children and families and any other service-learners or interns. She devised a pilot survey with three questions: two Likert style questions in which children could indicate using pre drawn facial expressions whether they liked making art or whether it improved how they felt. The third question, “What did you like to make?” could be answered through a drawing, with words, or could be completed by the parent. The ATRSL collected 16 of the “pilot” evaluations completed by child participants, assisted by art facilitators (i.e., artists or volunteers) who checked appropriate faces following participants’ verbal responses. All data showed “Strongly Agree” in the statement, “I liked making art during my visit”; 14 showed “Strongly Agree” in the statement: “I felt better after my art making sessions,” while 2 showed “Agree” in the statement. The free choice answers included drawings of symbols like heart shapes and flowers. Other answers were: “everything, scribbling, stars, sticker squares, or collage.” In addition to this survey, the ATRSL developed a survey for volunteers to ask about their hours, schedule and activities during volunteering, which was completed by three volunteers.

One outcome of this formative evaluation is the draft of the survey instrument and the student’s interpretation and recommendations of next steps. Recommendations of potential roles for the AHSL students were to recruit participants for the art making sessions, assist artists, provide art activities to the art groups; and otherwise assist or provide feedback to the volunteers. Recommendations were made to the Art Advisor as well, for example, meeting with volunteers before and after their sessions to provide information or ask for feedback for planning purposes. Recommendations to the ATRSL students for the next semester were to refine the survey, administer more surveys, and determine whether bedside art activities could also be evaluated for their anxiety-reducing potential.

One ATRSL began the second semester by observing one of the waiting room arts sessions and she also participated in creating art with the children. The ATRSL observed how the artist-facilitator introduced the project, which, for the particular session was a collage, and welcomed the children to join. There was no specific directive for the collage; but rather materials were available for children to construct a collage on a topic of their choosing. There was no discussion of children’s feelings regarding their illness or upcoming procedures, just art making. The children often joined and exited the group multiple times, if they were called to provide information to the nurses. After making art, the ATRSL observed that the artist-facilitator orally presented the questionnaire (developed in the previous semester) to each of the participants.

The ATRSL student found areas to refine on the 3-question survey including a place to add demographic data (i.e., gender and age). How children learned about the waiting room art making program, and how frequently they attended the art making session were also added questions. The ATRSL furthermore, reordered the questions so that what the children liked to make would be the second question. In the second semester, the questions were modified to be:

How many times did you make art during your stay?
I liked making art during my visit (Strongly Agree – Strongly Disagree).
What did you like to make?
I felt better after my art making sessions (Strongly Agree – Strongly Disagree).
How did you learn about this program?
Gender, Age.

This student also noticed the circles with facial expressions did not match the words, and she reordered the items so that the feeling and the image corresponded, e.g., so that “Strongly Agree” would correspond with a smiling face.

Forty-four completed questionnaires were entered into SPSS by the ATRSL student to calculate the mean, percentage, and the minimum/maximum of each category. Among the responses was that 59.1% strongly agreed that they felt better after making art and 38.6% agreed.

#### Soup Kitchen

The church-sponsored soup kitchen began an art program for children who arrived after school, about 2 h before the scheduled dinner serving. The staff referred to the art program as “food for the soul.” An art therapist designed and directs the art program, which is based on the values of education, creativity and imagination. The soup kitchen allows working families to come for meals to help them stretch their income.

Because of the unique nature of an art therapy program in a soup kitchen, the director is always looking for other similar programs that could provide ideas with which to enhance the programming. In addition to the art therapist, the art therapy program is supported by volunteers who sit at tables with the children as they make art. According to the art therapist,

everyone strives in everything we do to help our children love and care for themselves and the entire Earth community, think clearly, make choices wisely and learn alternatives to violence. Our emphasis on the arts is to stretch imagination, foster creativity and offer safe expression of feelings and values. The foundation of all we do is respect for the child and support for their families.

One student headed up the formative evaluation of the soup kitchen. She read program literature, attended a volunteer orientation, interviewed the art therapist, and attended an art therapy session. She learned that the art therapy goals and projects are planned in advance by the art therapist and volunteers. One of the adults at the table conveys the goal and instructions and if there are other volunteers they assist the children, or talk with them. The children are graded on the peace practices of creativity, courtesy and cooperation. Twice a year there is a “graduation” where the children are acknowledged for their participation and their “grades” for these peace practices. The director expressed a desire to know whether similar programs like her program exist, or if there was literature about how to teach peace to school aged students.

**FIGURE 6:**
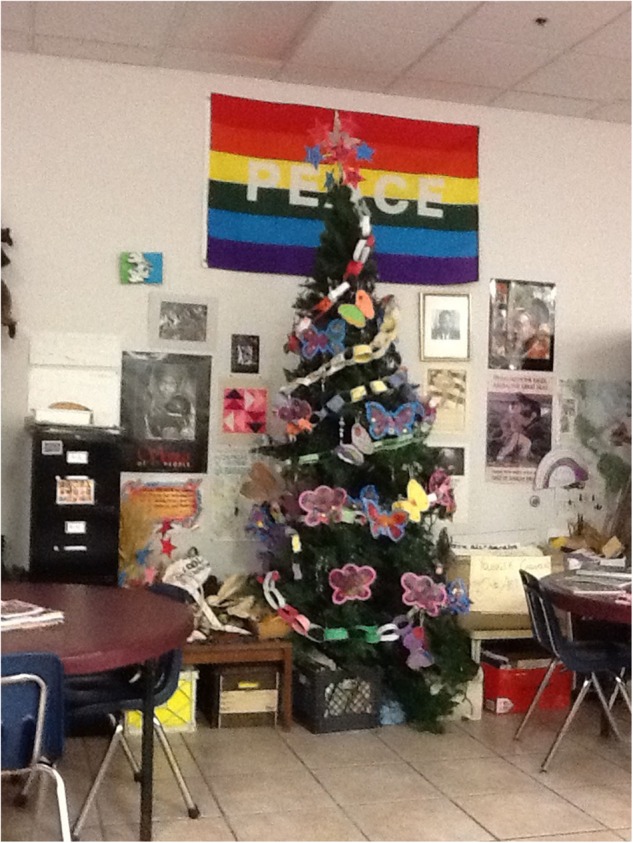
A view of the soup kitchen’s art room and peace tree.

Art History service-learners students in the subsequent semester’s class were recommended to emphasize the youth’s artistic creativity and assist with their art creations. The research students continued with the peace curriculum research. For example, in the second semester an ATRSL student completed a literature review on peace curricula, and also developed a program proposal for a peace curriculum (**Figure 6**).

#### Cancer Race

The Race to Cure Cancer is a city-wide race with approximately 40,000 participants that takes place annually to raise money for cancer research. It is associated with a cancer treatment center in which art therapy students have interned. The race takes place annually in the spring, which coincided with the second semester of our research study. On the 20th year of the race, the race director and staff wanted to commemorate the anniversary with a new dimension. Their vision was to offer a creative activity to race participants and their families that would allow race participants to creatively honor a loved one who had cancer, as well as to also offer a program for families with children who may be waiting for a family member to complete the race. The race personnel (agency partners) desired to enlist the help of arts service-learners to develop such a commemorative activity, but there were challenges to doing so that included determining a location appropriate for such an activity in the midst of high intensity pace of the race, no budget, and lack of storage for keeping any art created, after the race.

In the first semester, the ATRSL students conducted a formative evaluation to assess the resources and limitations involved in initiating a new annual commemorative event. Interviews were conducted with relevant personnel: the race (agency) director, the director of volunteers, and a longtime volunteer and the person who coordinated a children’s activity the previous year at the race. Additional data were collected through an email survey distributed through the cancer agency data base, observations of other fundraising events of the organization, a review of the agency’s published program materials, websites or practices of other US races. Finally, a review of literature on memorials such as the AIDS quilt helped to inform the project, and helped generate a list of potential ideas for the memorial art making activity. Ideas included a graffiti wall, sand mandalas, memory book with photos contributed by participants, group weaving, trees on which photos could be posted, performance art.

During the initial interview with race personnel, the authors posed the question, “What would you consider to be a successful event?” The responses suggested that success would be indicted if “Someone with a loss felt that they had a safe and special place to go experience the person’s memory;” Also, success would mean there was “a place for someone to think; a place to celebrate their life,” meaning both the lives of survivors and those taken by cancer.

**FIGURE 7:**
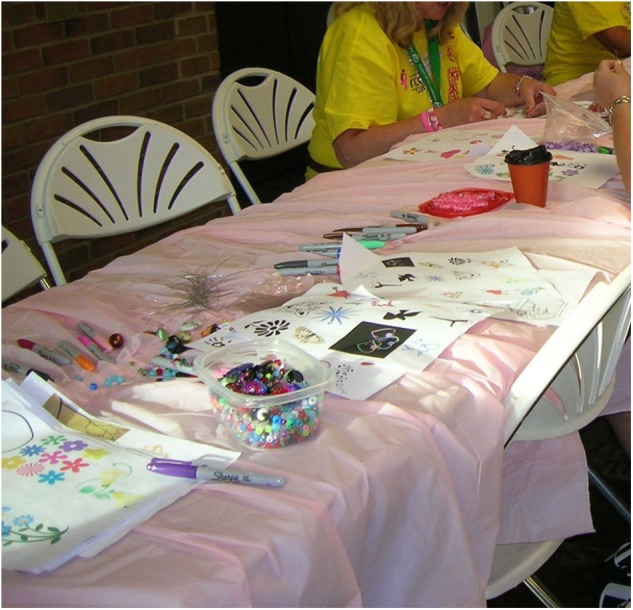
Making commemorative beads at the race to cure cancer.

**FIGURE 8:**
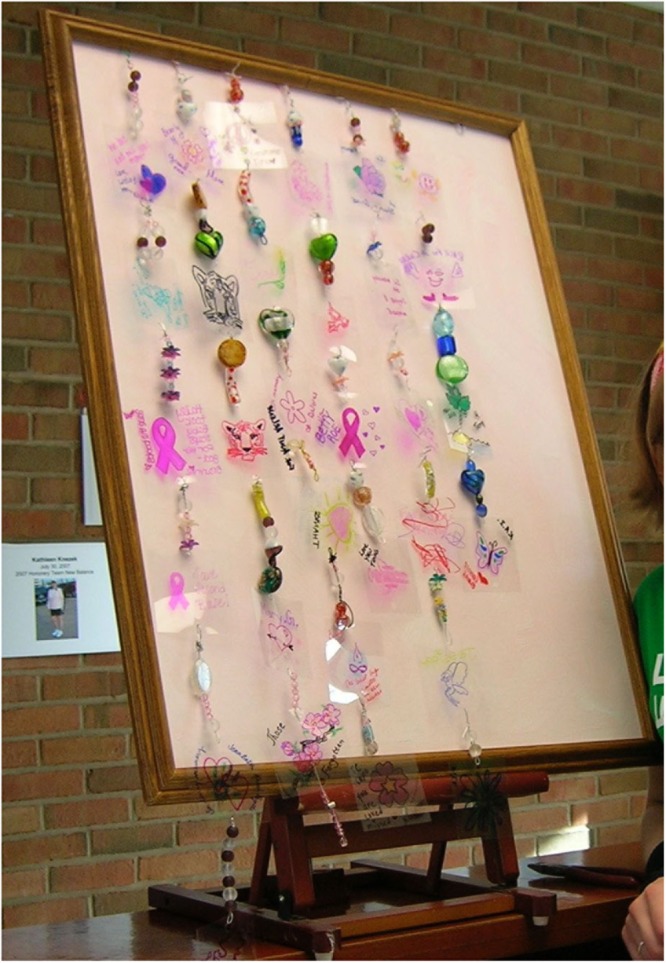
Cancer race commemorative art.

In the second semester, the formative evaluation was shared with the AHSL student who designed and carried out the commemorative event (**Figures 7, 8**). Due to the impending race schedule, the race personnel did not think they had a need for ATRSL students in the second semester. They chose to concentrate on the service-learner from the Art as Social Practice class who facilitated the creative activity desired. However, in an end of semester focus group, the race personnel expressed that the commemorative event seemed so successful that they desired to capture participants’ responses to it through an evaluation in the future.

#### Special Arts

The mission of Special Arts is to create an inclusive society where people with disabilities participate in, learn through, excel in and enjoy the arts. Special Arts addresses its mission in part through a program called Arts, Jobs, and Mentoring, which is a weekly after school program for teens located in two schools. Arts, Jobs, and Mentoring was the program for which Special Arts sought assistance. In this program youth participate in poetry/writing, visual art, music, and performing arts “to develop life enhancing and pre-vocational skills that prepare them for entering the world or work…” Special Arts was looking for methods to identify how the Art, Jobs and Mentoring program impacted their participants.

Two ATRSL students reviewed the Arts, Jobs, and Mentoring operation and assessment practices, and explored research methods that could enhance their current efforts to determine whether their goals were being achieved. The ATRSL students interviewed of the director and the two teachers of the programs in each of the two schools. They read program descriptions, and observed a class. The researches wanted to interview school personnel or survey students and parents, but this was not possible.

The data they collected revealed that transportation was a problem at one school, impacting participation and attendance. Furthermore, both schools collected data but did nothing with the data. The researchers looked for a theory of program-process that would help to reveal the problems preventing analysis of the data, as well as other in-process data collection methods that might be used for program improvement.

This formative evaluation study researched three perspectives on how to achieve Special Arts’ goal of measuring the personal development of students participating in the Art, Jobs, and Mentoring program. Secondly, to capture the strengths of the artistic elements of the program, the ATRSL students planned to further investigate how other community arts programs achieved program evaluation.

In the second semester, multiple factors resulted in no AHSL student being placed at Special Arts. Had there been a student with a strong interest in the program, Special Arts would have accepted the student, however, this was not the case. Also, the director had decided to retire, and the agency was consumed with making plans following the retirement.

In order to assist Special Arts as much as possible, they were offered the assistance of a doctoral student from the university who had the kind of statistical expertise that Special Arts desired. In addition, an ATRSL completed a literature review on Assessment of Community Art Programs, and a copy of this review was provided to Special Arts.

## Findings/Results

The findings are organized by the three research questions:

**What are students’ experiences as service-learning participants, and what are the learning outcomes of their service-learning experiences?**

Sixteen total art therapy students enrolled in research courses in the two semesters, including three who were in both semesters’ classes, despite expecting the same students to register in each of two consecutive semesters. Twelve consented to be research participants. Excerpts from the questionnaires are displayed in **Supplementary Data Sheet S2**.

The students demonstrated willingness to learn research as research participants themselves. They learned methods that would have been taught in a non-service-learning research class, plus they were able to practice the research methods with real agencies, which was according to students, “exciting” and “broadened horizons.” One student wrote, “the more I learn, the more I gain,” and another wrote,

Basically, I’m surprised by the interesting challenge of the master’s project. I don’t think I would have attempted this research on my own so I am grateful for being thrown into the deep end and excited to have the chance of possibly providing a valuable service to an organization.

The research products were also evidence of students’ research knowledge gained; in particular, program evaluation. One student wrote, “Learning about the components of program evaluation has given me ideas about how to look at programs critically, but not as a critic.”

The depth of student learning is exemplified by this comment:

I’ve learned that defining goals and objectives clearly and knowing the purpose of the research is key in collecting useful data… It seems that through research the providers of a program can clarify its purpose and impact, but the trouble is most arts-based organizations don’t have the time, staff, or resources to implement their own research. Something I have realized through the reading in the class is how essential the theoretical model of an organization is as well as the theory behind the research being conducted. I also have become more cognizant of collecting process data and how it can enhance and breathe life into outcomes data.

Several mentioned that this experience might be useful to them in the future if they were in the position of managing an art therapy program. One student indicated the value of interacting with volunteers or others who might be able to enhance an art therapy program.

One student acknowledged the importance of community research, that “we cannot focus on the lab only.” A second commented that “although the amount of research we did was minimal, it may have a powerful impact on the community partner.”

Service-learning fostered personal growth: Two students wrote about learning how to work with frustration, and “how to stick with doing things and doing your best even when you didn’t want to.” One student wrote that because of the stress induced by this particular course, the student “… learned more than research. The most important thing I learned was that ‘it’s actually ok to ask people when you need help.”’ One student was “nervous about the whole interview process [but] I think I handled myself well.” Another acknowledged “the experience influenced me to pursue grief and loss as a focus area.”

Service-learning contributed to both positive and negative personal awareness through working with others. Two positive comments about partners were that working with a partner was enjoyable, and made it easier to brainstorm “by having someone with whom to discuss my findings or interpretations of data.” Community skills, cooperation skills and compromise were also mentioned. There were difficulties noted with working as a team and the distribution of work load. One admitted not being easy to work with. One student did not “feel a connection” to the other student partner, but did feel a connection with the agency. Others wrote that the effort and time involved was more than expected and they were challenged with time management in spending time in the field with working and going to school.

There were equal numbers of opinions for and against the way the class was organized. Some students did not like pursuing a research agenda not of their own choosing, whereas others “liked that the topics were given to us to choose from rather than finding my own topic.” One student wrote,

I was excited. I willingly dropped my original master’s project focus to dive into something that was hopefully and going to provide useful information to serve a valuable purpose for an arts-based non-profit organization… Do I feel I am doing something worthy? Yes.

**What are community arts organizations’ experiences with service-learning students, and how does the involvement of service-learning students affect the overall effectiveness of their programs?**

During the final focus group all of the agency directors unanimously agreed that the formative research performed throughout the first semester increased their awareness about art-based service-learning. Furthermore, the first semester’s research activities helped the agencies prepare for the subsequent semester when they had the additional AHSL students.

The agency directors also acknowledged the benefits of the research to their agencies: The research students helped to create new program elements or increased the diversity of programs offered; they helped to identify or develop new resources and helped serve the patient population. The directors enjoyed the mentoring relationship and they commented on the personal qualities of the ATRSL students including excitement, enthusiasm, new ideas, knowledge, a youthful point of view, sense of optimism, and belief in creativity. Despite the benefits, students cost agencies primarily in terms of time. A more detailed list of benefits is provided in **Supplementary Data Sheet S3**.

In the first semester, the agency directors answered questionnaires aimed at determining what kind of service-learner they desired, and also the types of projects with which the service-learners could expect to assist. Personal qualities were important to a successful service-learning relationship. The qualities directors said they looked for in students included motivation/passion, responsibility/commitment, independence, creativity, emotional intelligence, leadership/ability to be a role model, flexibility, preparedness.

Although the specific projects undertaken ideally are negotiated between the agency and the individual service-learner in order to engage the student’s strengths and abilities, the university researchers hoped that by developing a list of potential projects, incoming ALSL students could begin thinking about the possibilities before the semester they were enrolled, possibly allowing them to begin their work right at the start of the semester. This was a small concession toward the problem expressed by agencies: that the time necessary to orient and train new students leaves insufficient time in the same semester for the students to contribute, leading agencies to feel that students’ contributions are not worth the agencies’ investment in the service-learning relationship.

We did not completely solve the problem about service-learning’s connection to a single university semester. Based on our reflections and discussions in our bi-weekly research meetings, as well as focus group discussions, possible resolutions for scheduling problems and suitability of fit included: (1) development of clear expectations for service-learning students and agencies, (2) improved assessment of strengths of students in order to suitably match with agencies, (3) more opportunities for feedback in order to improve communication between students and agencies, and (4) establishing incentives for service-learning students to continue on in agencies to establish consistency and continuity.

### Assessing Program Effectiveness and Outcomes

This article has been focused in large part on describing how the research service-learners “supported community partners to assess their programs’ effectiveness” through formative evaluations and then through summative evaluations or other culminating projects useful to the agencies.

As stated, the ATRSL students attempted to respond to the needs of agencies for continuity of services over more than one semester. The research students continued their presence at the agencies in research capacities, but also as liaisons between the agencies, and the AHSL students in the second semester.

The research undertaken at Recovery House and the children’s hospital, especially, have allowed for the collection of data from community participants (the constituents of the agencies) which helps to inform art therapy practice in terms of how art making is experienced by individuals.

At Recovery House, the project assessed was building a mosaic with dominoes using a grid, something that could hardly be considered art making. However, the resulting product was displayed as art and there were technical skills and process decisions necessary to accomplish it. What the project seemed to do was to foster the team building that the program desired. Perhaps not unlike the old-fashioned quilting bee, working in close proximity with other recovering persons enabled talking to one another and learning about one another. Another outcome of the *Yes, We Can* mosaic was that the men were proud of their co-creation; proud of its message to other recovering people, and proud of being in the recovery community. Collective self-esteem (Taiffel and Turner, 1986) is defined by the way a person’s concept of self is derived from both personal identity and social identity – or, that part of individual’s self-concept, which is based on membership in a group, and the emotional significance attached to that group. The men seemed to develop a sense of collective self-esteem about being a member of a recovering community, and they desired to share their knowledge of recovery with others.

At the children’s hospital, the 44 collected questionnaires showed that 59% of the children strongly agreed and 38.6% agreed that they felt better after making the art. Both female (25) than male (18) children participated, ages 1–17. Twenty-two of them were ages 7, 8, or 9. These are some basic descriptive data about therapeutic art participation that allow for a deepening line of inquiry, such as, what about the art making made you feel better? How do you describe feeling better? These are the types of questions that could be pursued in subsequent semesters. Furthermore, service-learners could observe and collect information about the kinds of art projects that produced the most favorable results.

## Discussion

The value of qualitative data is that they focus on naturally occurring, lived experiences, provide rich descriptions on what life is really like and the meanings people attribute to their experiences (Miles et al., 2014). Sometimes surprises lead to important follow up. For example, we were surprised that some students had difficulty working together; we assumed that the art therapy graduate students would be supportive of one another and have the interpersonal skills that would enable partnerships. Although most seemed to, not all did. Thus, it may be important for instructors not to make assumptions about students’ abilities to work in partnerships with others, but rather to include a discussion about expectations, and work to help students develop interpersonal skills. Recovery House wanted students to assess whether or how a group art project facilitated teamwork. It is important that student researchers have some awareness of their own abilities to function as team members. How can professors foster community and relationships in a classroom? How can graduate programs facilitate interpersonal connections among its student body? If fostering community is so important, should art therapy educators do a better job at this ourselves?

The question of how students experience service-learning was intended to pertain to all the service-learning participants, such as those AHSL students. Because the ambitious research responsibilities consumed much of the time for reflection, this may have impeded the experience of the ATRSLs. It could be that students did learn more than they realized, but they might have learned even more with consistent encouragement to reflect on their knowledge construction and affective development. By definition, the research students had a service-learning assignment: they provided a service to the community that was valuable as well as relevant to the course description, yet without the consistent reflection, we believe the students missed an important component. By the end of the semester, when the students gave their presentations to the agencies, and all the students heard the presentations of their colleagues, some students remarked on the gratitude they felt to have been part of this research project. They finally seemed to recognize the meaningful work that they did. We wished we could have been more effective in cultivating a sense of activism, better balanced with managing all the other responsibilities during the semesters.

Although one motivation for engaging the research students as service-learners was their anticipated registration in two consecutive semesters, which would have allowed for continuity of presence at the agencies, as it turned out, not more than three students registered for both of the research courses. Yet, because of the effort to ensure continuity through ongoing projects, through mentoring the incoming students at the agencies, this seemed to help achieve that sense of continuity, and reduce the burden of time to orient the new students. Still, relationships are individually made, and this dilemma continues to be explored.

Community engaged scholars (McNall and Barnes-Najor, 2018) assert that student learning outcomes and community outcomes associated with CBR are largely unchartered territory. This article begins to navigate that territory. This study demonstrated a process to research that sought practical knowledge desired by the community partners. Watkins and Shulman (2008) ask researchers to reflect on whether the work we do mirrors our dream for a community or the community’s dream for itself? Our research mirrored both dreams:

For the community, the university partnership provided outside perspectives on community strengths and helped identify places where service-learners could assist communities with realizing their goals. Although we did not continue with the service-learning assignment in the research classes, this shift in attitude is something we have endeavored to foster in other field based classes. That is, to approach art therapy practice less as the expert, and more as a student of the community agency, receptive to what the community can teach the university constituents. Students also begin to think less about what the agency experience can add to their resumes and more about how they can contribute to and support the agencies’ goals.

The focus groups allowed agency directors to meet each other and support each other. In fact, the agency directors also requested ArtsCorps to continue to share any future opportunities for the partner agencies to collaborate with one another.

Once, one of the agencies announced a fundraiser and in a subsequent meeting someone from another agency donated a gift basket. Small gestures like these seem indicative of community building. More importantly perhaps, like the sense of collective self-esteem we believed we observed at Recovery House, something similar seemed apparent in the focus groups. As the agencies talked about their services, they did so with the sound of pride. Their own sense of identity as an arts program seemed bolstered by membership in a group of similar agencies.

The relationships between the university and the agencies continue today, and additional opportunities have developed from our relationships: Art Street was able to hire one of the AHSL students as a curator, who then was instrumental in mentoring ArtsCorps volunteers. Art Street and the Art Therapy program have applied for grants together. Currently Art Street is planning a conference studying its 30-year history to explore its contributions to its constituents and to the city. Art therapy research students will have key roles in participant observation and recording of the breakout sessions.

The children’s hospital continued to study the waiting room art program. The ArtsCorps pilot led to approval of a hospital IRB, and a third research student who collected 150 surveys from children and parents, and the research continues.

Recovery House established an art therapy position, and the art therapist mentors students in service-learning and internship capacities. The ArtsCorps graduate assistants helped write a successful mini grant for the Soup Kitchen, which provided for programming for their peace camps, and they hired a second art therapist. The Special Arts program continued to accept interns, as does the Cancer Center, which also hired another art therapist. The cancer center’s creative/commemorative event grew with subsequent years’ service-learners. For example one student worked with girl scout troops and other community groups to make hundreds of life size cardboard figures, all painted pink for breast cancer awareness, and they were placed along the race route, with names of loved ones. The Cancer Race now desires to assess the impact of the commemorative art making on those who participated.

For 5 years, the ArtsCorps program collected data from arts service-learners; and with a total of 11 agency partners who continued to participate in focus groups on mutually beneficial service-learning programming. We collected data from four art therapy introductory classes, and two music education classes. These findings were reported in Feen-Calligan and Matthews (2016). Although we have closed the research study, we continue to have service-learning assignments in the introductory art therapy classes.

The agencies’ interest in evaluations led to the idea that ArtsCorps could sponsor weekend evaluation “camps,” staffed by service-learners with expertise in urban studies, social work and psychology who together could work with agencies to develop logic model evaluation plans. We successfully applied for grants that allowed us to offer two sessions of “Camp Evaluate” which were attended by two of our original research partners as well as new agencies.

As Harkay et al. (2000) write, it important to reflect on what we learned and how we could do it better. Future research could benefit from thorough planning the assessments, time and organization of the data collection, survey design and mixed methods. The study suggests that art therapy programs might benefit from teaching program evaluation and outcome evaluation of community art programs.

In summary, students gained research knowledge and skills particularly in program evaluation, a form of outcomes-based research, and they learned about agencies in greater depth than they would ordinarily have learned in experiences like internship. Knowledge of community should serve their art therapy practice. Furthermore, whether they realized it at the time, the opportunities to practice flexibility, negotiation, tolerance, and listening are good preparation for working with others.

## Ethics Statement

This study was carried out in accordance with the recommendations of Wayne State University Internal Review Board, Human Investigation Committee. The protocol was approved by the Human Investigation Committee. All subjects gave written informed consent in accordance with the Declaration of Helsinki.

## Author Contributions

All authors listed have made a substantial, direct and intellectual contribution to the work, and approved it for publication.

## Conflict of Interest Statement

The authors declare that the research was conducted in the absence of any commercial or financial relationships that could be construed as a potential conflict of interest.

## Supplementary Material

DATA SHEET S1 Art History: Art as Social Practice student service-learning projects.

DATA SHEET S2 ATRSL student post-service-learning questionnaire.

DATA SHEET S3 Findings from agencies.
