# Supplementary material for: Art Therapy, Community Building, Activism, and Outcomes
Source: Front Psychol. 2018 Sep 24;9:1548. doi: 10.3389/fpsyg.2018.01548 (PMC6176654; doi:10.3389/fpsyg.2018.01548)
Supplement: DATA SHEET S1 — Art History: Art as Social Practice student service-learning projects. [file Data_Sheet_1.pdf]

AH: Art as a Social Practice

Student Community Placement Activities

Children's Hospital
AHSL 1: Design patient friendly rooms for the handful of treatment rooms assigned. Since each floor consists of a theme (4th floor is garden, 5th floor is sky, etc.) concepts for the designs are based on these themes. Through the designs it is hoped the patients who must visit the treatment rooms will be less scared

AHSL 2: Develop some original artwork for them, i.e. creating art for one of the treatment rooms. Also work on two logos: one for a healthcare conference coming to the city hosted by the hospital and another for the bedside art program. Both logos will be in multiple digital formats with one of them also being animated.

AHSL 3: Work in the "HEALING ARTS PROGRAM" to provide projects and programs to help patients and their families by providing relief from anxiety and boredom, distraction from pain, and a creative outlet for expressing emotion. Create a parental guidance system for the families visiting the hospital, which it will be similar to a map directing parents to different artworks or activities throughout the three different floors, at the same time the guidance will some type of a game to where the parents and the children will be guessing which piece of art it is that's indicated on the map.

AHSL 4: Do arts and crafts with children in the clinic waiting room. They are not in-patient, so the projects are just quick ten-to-fifteen minute projects to keep the occupied and allow them to do something creative. The hope is also to come up with some kind of long term project that would involve various works the children create, or something the hospital can use in the future. Also designing projects that are not just art but can also be used in the child's everyday life (such as bookmarks or picture frames). Overall, the hope is hope that children to have some comfort while at the hospital and to do something creative in that short time.

AHSL 5: Participate in the camp for the kids. Participate in the treatment rooms doing some artwork with some of the kids admitted in the hospital to make them more comfortable during their stay.

Recovery House
AHSL1: Assist with the process of working to assemble a portrait of Obama with dominoes. Photograph the process.

AHSL 2: Assist with the domino project in which likeness of President Obama is reproduced because it to encourage social interaction. This project teaches the members how to work together while building their self-confidence.

Art Street:
AHSL 1: Assist with the “Street Folk” project including painting 16,000 shoes to represent 16,000 homeless people in the city. Each shoe is going to come with a story, Sort and read stories to decide which ones we will be using to photograph for the book (See Figure 8)

AHSL 2: By reclaiming discarded items and transforming them into art objects, Art Street offers the community an avenue for creativity and hope in the face of adverse conditions. This year is the 25th twenty-fifth anniversary year of Art Street; many events are in the works to celebrate their achievements and promote their ongoing work. Work with the development officer on grants and development and other ways to mark the anniversary.

AHSL 3: Construct models for the artist for a project that the artist is planning utilizing the Brewster Buildings.

AHSL 4: Work at the Art Street and the African American Museum exhibit. Do research to find some archival pieces for the show. Look through storage at the Art Street offices to find some more three-dimensional pieces for the show.

AHSL 5: Work with the Art Street Office to help the artist with various graphic design related assignments to help promote some of the things he has coming up

Cancer Race:
AHSL1: Create some kind of interactive memorial for during the Race. Consider ideas from fall students that have to do with beads. The idea is to have plastic squares that can be strung on wire or string available by small donation on the day of the Race. There will be permanent markers in many different colors set out on a table so people can write a person’s name or a message onto the bead. Try to get a quiet location on the day of the Race so the people who come can quietly reflect while they design their piece(s). Accrue supplies (supplies, volunteers, etc.), when we need it, and how the organization of the craft itself and develop flow chart.

Soup Kitchen
AHSL 1: Read picture books to the younger kids and give them a drawing / illustration assignment which will go along with the theme of the book. Also conduct basic figure-drawing workshop with a group of High School students who have an interest in honing their own skills in drawing. The main focus of these classes would be drawing from direct observation. The students would take turns modeling for each other. We will explore different techniques that are essential to the foundation studies of a young artist. Topics and Techniques to be covered:

- Gesture Drawing
- Breaking a figure down into basic shapes
- Using a sight gauge
- Line quality
- Facial structure
- Constructive critique etiquette.

AHSL 2: Work the children at the Soup Kitchen. The project will be within the after-

school programs for kids located in the Children's Library inside the soup kitchen. Organize a drum workshop where I teach kids to make and decorate their own drums and learn the rhythmic qualities of drumming. The course will include videos of drummers, lectures on the origins of drumming in many different cultures, and, of course, making their own drums out of oatmeal containers, rubber-bands and balloons. Kids create and design their own drums first and then as the class progresses maybe bring in actual professional drums and percussion for the kids to play. On the final day I will bring in some recording equipment and record a song with the kids that they will help write.
