# Supplementary material for: Art Therapy, Community Building, Activism, and Outcomes
Source: Front Psychol. 2018 Sep 24;9:1548. doi: 10.3389/fpsyg.2018.01548 (PMC6176654; doi:10.3389/fpsyg.2018.01548)
Supplement: DATA SHEET S2 — ATRSL student post-service-learning questionnaire. [file Data_Sheet_2.pdf]

Graduate Research Students

Post- Service-Learning Questionnaire

Answers to Select Questions

Course Enrolled	Date	Expected graduation date	Name	Access ID

Did your opinion about research change as the result of your research class(es?) If yes, how?

No, no

A lot more difficult than other research classes

Yes, I've opened my eyes to the variety of ways research can be done

Greatly increased -better grasp of theory and precedents in service-learning research

Much harder than I thought

Its more interesting and potentially more difficult to research someone else's question

Did this service-learning research class contribute to your knowledge of research? If so, please elaborate.

Yes, I did not realize the amount of work and effort that goes into the work.

Yes, I feel community is important to research, we cannot focus on lab only

Yes, it reinforced that ABR is important

Yes, I learned that although the amount of research we did was minimal it may have a powerful impact on the community partner

Yes, it's challenging to work with a partner

Yes, I did not know either summative or formative evaluation

Yes, I have more awareness of the possibilities and complexities involved in research-I look at research articles more critically

Yes, very much. I understand the process and how it can help validate my work as a professional

Did this service-learning research class contribute to your knowledge of your discipline (e.g. art therapy/theatre?) If so, elaborate.

Yes, I thought of doing this type of eval for grants and other endeavors but did not know

how

Yes, I know more real world; I gain experiences in both academic and practical ways

The class helped me realize the students are not necessarily the only party to benefit from the service-learning relationship

Yes, I'm interested in assessment. The research I did helped me understand how I can implement service-learning in the class and how to use reflection as assessment

Not particularly; I wasn't able to get into any aspects of art therapy with the participants

Not really

It did expand my knowledge somewhat of art therapy as a profession

Yes, particularly about accessing research and data

Yes, art experientials aren't always equally enjoyable nor applicable

Art collections have a history and may be controversial

Possibly-I may be able to use some of these processes that I used to assist my service-learning organization with some other organization that I may work for

Did this service-learning research class contribute to your personal growth?

E.g. to your ability to work with others?

Yes

I learned I can be difficult to work with

Yes. Partnered up to visit the site by a classmate and worked throughout the semester, sharing work, etc.; a good experience

I felt I didn't have a connection with my partner but I did have a connection with the agency

I think I work pretty much the same

Communication skills?

Yes, I was nervous about the whole interview process and I think I handled myself well

Yes, yes

Yes, I got a chance to work with a partner, I could brainstorm better by having someone to discuss my findings or interpretations of data with.

I kind of dropped the ball in working with my partner

Made me realize I need to work on this; make improvements be more timely

Learned to be more direct and to the point

Problem solving?

Yes

How to budget time

Yes, through the assignment and its unique research qualities

I’ve learned sometimes you just have bits and pieces and you have to connect the pieces

Other personal growth areas?

Searching articles/data abilities; coping with pressure better at keyword searches

Standing my ground and trying to reach a compromise

Recognizing internal skill strengths and weaknesses

Time management I need work on

Need more outlets for stress

It helps validate my professional ideas

If you had concerns about this class initially, how were your concerns resolved/addressed?

Initially I wanted to work on my own research questions but instead was able to learn some approaches that I could use for data collection in the future.

I wasn’t sure what we were researching initially but with my partner we found the answers

I wasn’t sure I could apply the work I was doing to my work. I had to find those answer for myself by doing the research.

What did you learn in this class?

There are multiple ways to conduct research

A lot! Research skills, community skills, cooperation skills. I’m very young in this field

I got a refresher on the scientific process, learned data collection, got an idea of how to approach a community partner to conduct research and was able to write a formative evaluation

I learned that research is not quite as easy as I initially thought Also that when working in a group compromise Is key to working with other.

How to stick with doing things and doing your best even when you didn't want to- how to work with frustration

I learned everything I know about program eval; a lot about data collection

Research doesn't have to be boring

Formative eval not for me

How to do research and apply to real life issues

Pre and post responses to rating commitment to serve others

and commitment to service to the profession

Student	1	2	3	4	5	6	7	8	9
Pre Commitment to serve others	4	5	4	4	5	4	5	4	5
Post Commitment to serve others	5	5	4	5	4	4	2	5	5
Pre Commitment to service to profession	4	5	4	5	4	3	5	5	5
Post Commitment to service to profession	5	5	5	5	5	4	4	5	3

1=no commitment 5= very committed
