# Supplementary material for: Art Therapy, Community Building, Activism, and Outcomes
Source: Front Psychol. 2018 Sep 24;9:1548. doi: 10.3389/fpsyg.2018.01548 (PMC6176654; doi:10.3389/fpsyg.2018.01548)
Supplement: DATA SHEET S3 — Findings from agencies. [file Data_Sheet_3.pdf]

ArtsCorps Summary of Findings from Agency Director
Pre, Post, Interview, and Focus Group Data
Fall - Winter Semesters

<u>Qualities the community agencies look for in service-learning students</u>

Intrinsic traits:

- Motivation/passion
- Responsibility/commitment
- Independence
- Creativity
- Emotional Intelligence
- Leadership/ability to be a role model
- Flexibility
- Preparedness

Competencies:

Skills pertinent to the site's needs such as:

- Art techniques
- Computer proficiency (ex: web design, internet research, media development)
- Research
- Communication
- Problem solving

A student who can help with:

- Stand alone or ongoing projects
- Providing services/programs to client population
- Designing, collecting, and analyzing data for program evaluation
- Administration
- Planning and organizing
- Web and media support

A student who is prepared with the knowledge of:

- The history, mission, goals, and programs of the agency
- The importance and role of art in the city
- The populations served by the agency
- How art helps their client population
-

Specific benefits of service-learning identified by the 5 agencies

Cancer Race

- Provided "non-monetary" benefits such as "the gift of time."
- Focused on specific client population and developed new program element to serve them
- AHSL student created a lasting, re-usable project

Recovery House:

- Helped create an art therapy program at the agency
- Created art projects in collaboration with population served by agency
- Collected and analyzed data

Soup Kitchen:

- Resource development
- Partnering and networking with local agencies
- New ideas and increased diversity in programs offered
- AHSL student located local talent/guest artists

The children's hospital:

- Acted as an "unpaid workforce" supporting a small department
- AHSL student created a tangible outcome [public art] for the program
- Developed materials to support ongoing programs
- Helped serve patient population

Connections enhanced and/or further developed by service-learning (as reported by agency directors)

- Connected community members with art opportunities
- Students provided a link between agency and community (by individually reaching out to community members)
- Increased relationship between agency and university art therapy program
- Agency felt increased connection with other local social justice and art groups/agencies
- Enhanced connection between service-learning and community through public/visible projects
- Networking took place between the agencies involved in ArtsCorps research
- Service-learning connected agencies with external resources such as grants/funding, media/publicity, and exhibition space
-

Service-learning challenges from the agency perspective

- Scheduling
- Student's lack of free time
- Student inexperience
- Student lack of follow through
- Difficulty identifying economic benefits of hosting service-learning students
- Strain on agency resources (take up space, use equipment, some require supervision)

Potential solutions to service-learning challenges

Before placement:
Agencies and University could:

- Brainstorm how to best use service-learning students at their agency
- Recognize that service-learning students often have different motivations and needs than volunteers
- Have service-learning students submit a short bio and resume to agency prior to placement
- Exchange student/agency schedules and check for compatibility before placement, and then develop a consistent schedule for service-learning communication/activity
- Determine a timeline for email/phone/and in person contact between student and agency
- Clarify expectations at the outset (ex: not accept students who cannot make the time commitment required), and communicate guidelines for successful service-learning

Students could:

- Reflect prior to placement by identifying their value system, strengths, and interests (through use of inventories and assessments)
- Identify and base their work on why art matters to them and how they find art healing
- Identify their preferred work style (ex: team vs. independent, ongoing vs. single project, client contact vs. no client contact)

During placement:

- Increase mentoring of students by university, agency directors, and agency staff
- Choose projects that are efficient and realistic based on agency and student's time and skills
- Increase documentation and reports evaluating student's performance (in process/mid-semester evaluation)

Ongoing:

- Create service-learning handbook/s (manuals for agency and student to include guidelines, evaluation forms, important information)
- Have ArtsCorps staff member act as placement coordinator, serving as a liaison between student and agency (prepare agency and students, place students more effectively)
- Encourage longer-term relationships between agency and student
- Develop incentives for students to continue work with agency (offer directed study, one credit course, stipend)
- Increase off-site training of students

Questions to Consider

- Is the orientation process in the classroom and at the agency the same as volunteer orientation or more specific to service-learning?
- How does the agency's schedule compare to the student's schedule?
- What are the past successes and failures of service-learning at the agency and how can the experience improve for all participants?
- What are the individual mentoring needs of the student?
- What can realistically be accomplished (what resources and time are available for the project)?
- Is the student personally connected to or inspired by their mission/reason for choosing their site/project (what kind of service, why, and where)?

Student Findings

What are students' experiences as service-learning participants, and what are the learning outcomes of their service-learning experiences?

Data:
Pre S-L course baseline survey (9 of 14 responses)
Reflective blogs written during service-learning work (14)
7 Post S-L course surveys
Questionnaire/interview data from 7 alumni from 2008-2010
6-month post course survey to be administered October 2011

•Based on blog data and pre/post survey data, individuals in W 2011 class experienced

- Inter and intra personal learning
- Personal growth, e.g.: Learning about themselves and how they interacted with others often from different backgrounds than their own (Diversity learning)

- Increased sense of self-efficacy to contribute to change in the City of Detroit (Democratic citizenship learning, Social responsibility learning)
-Learned about *social* role of the artist in society

- Increased intrinsic motivation (6/7 stated plans to continue to volunteer) (NOTE: Alumni participating have all continued their community service in some way.)

-Learned about their future profession
